# Fine Structure of Posterior Alpha Rhythm in Human EEG: Frequency Components, Their Cortical Sources, and Temporal Behavior

**DOI:** 10.1038/s41598-017-08421-z

**Published:** 2017-08-15

**Authors:** Elham Barzegaran, Vladimir Y. Vildavski, Maria G. Knyazeva

**Affiliations:** 1 Laboratoire de recherche en neuroimagerie (LREN), Department of Clinical Neurosciences, Lausanne University Hospital and University of Lausanne, Lausanne, Switzerland; 2 Leenaards Memory Centre and Department of Clinical Neurosciences, Centre Hospitalier Universitaire Vaudois and University of Lausanne, Lausanne, Switzerland; 3 Department of Psychology, Stanford University, Stanford, CA USA

## Abstract

Heterogeneity of the posterior alpha rhythm (AR) is a widely assumed but rarely tested phenomenon. We decomposed the posterior AR in the cortical source space with a 3-way PARAFAC technique, taking into account the spatial, frequency, and temporal aspects of mid-density EEG. We found a multicomponent AR structure in 90% of a group of 29 healthy adults. The typical resting-state structure consisted of a high-frequency occipito-parietal component of the AR (ARC1) and a low-frequency occipito-temporal component (ARC2), characterized by individual dynamics in time. In a few cases, we found a 3-component structure, with two ARC1s and one ARC2. The AR structures were stable in their frequency and spatial features over weeks to months, thus representing individual EEG alpha phenotypes. Cortical topography, individual stability, and similarity to the primate AR organization link ARC1 to the dorsal visual stream and ARC2 to the ventral one. Understanding how many and what kind of posterior AR components contribute to the EEG is essential for clinical neuroscience as an objective basis for AR segmentation and for interpreting AR dynamics under various conditions, both normal and pathological, which can selectively affect individual components.

## Introduction

The most prominent activity recorded with electroencephalography (EEG) from the scalp of a healthy human at rest is the near-sinusoidal oscillations within the 7–13 Hz range called the alpha rhythm (AR). Multi-rhythmicity of this frequency band has been known for a long time^1, 2^. Empirical evidence shows that the waking AR consists of several oscillatory components, which differ in scalp topography and reactivity to sensory stimulation or to motor activity. The family of AR includes the central mu rhythm, the tau rhythm, recorded over the temporal cortex, the anterior AR, and the widespread and ample posterior AR^2, 3^. The latter, also termed the occipital or visual AR, is either considered a unitary phenomenon^4–6^ or divided into 2 or 3 sub-bands^7–11^.

By and large, the authors of EEG studies assume the heterogeneity of the posterior AR as a well-established fact, based on which they demonstrate individual properties of preselected AR sub-bands under various physiological and pathological conditions. A non-exhaustive list of supporting evidence includes: different responses of the low- and high-frequency ARs to visual stimulation^11–13^; frequency-specific AR correlates to memory and attention^14, 15^; the selective efficiency of the high-frequency AR in neurofeedback training^16^; and separate alterations of the low- and high-frequency ARs due to neurodegenerative processes in old age^8, 17^.

Although the above findings suggest that the surface-recorded posterior AR represents a combination of rhythms, their number as well as their cortical sources require a systematic investigation, which would involve an analytical determination of AR components (ARCs) based on EEG spectra obtained with a high frequency resolution. Along these lines, Chiang and colleagues^18^ studied the AR structure in 1500 healthy subjects by means of cluster analysis. Of all participants, 44% showed a two-cluster AR, while in the rest of the population, multiple peaks remained unresolved. Another study^19^ based on a similar approach identified multiple ARCs only in 1.6% of 1215 subjects. The inability to separate ARCs in many EEGs can be attributed to the methods that took into account the visibility of separate spectral peaks, but underestimated the spatial and temporal dynamics of the AR because of crude spatial sampling (19 electrodes) and short EEG recordings (2 min).

Alternatively, factor analysis was applied to the EEG power of healthy subjects^20^ and patients^21^. In both studies, 2 independent ARCs, which, in general, corresponded to empirically determined low- and high-frequency ARs, were reported. However, being a bilinear decomposition technique, factor analysis cannot fully account for the multi-way nature of EEG data. Specifically, the spatial aspect of AR was ignored since only a few preselected EEG derivations were used. Moreover, these attempts of AR decomposition were based on short recordings of low-density referential EEG, thus leaving beyond the scope of analysis the sources of ARCs, as well as the extent of their independence, stability, and dynamics at various time scales.

This motivated us to test the hypothesis that the posterior AR in healthy young adults has a structure characterized by separate components with individual spatial, frequency, and temporal features with advanced techniques involving a multi-linear decomposition of mid-density EEG. Considering the assumed role of the AR in the mechanisms of cognition and its dramatic changes during human development and aging, detailed knowledge of AR component structure can help us better understand the role of oscillatory activity in information processing and develop efficient biomarkers of the brain functional state.

## Methods

### Subjects

Thirty-three healthy subjects between 20 and 45 years old were recruited for this study. Here we report the data from 29 of the subjects, since EEG data from 4 subjects were excluded from the analysis owing to the excessive muscle artifacts (1 EEG), no visible AR (2 EEGs), and the interrupted recording session (1 EEG). The group consisted of 17 men and 12 women (mean age 31.6). Prospective participants underwent a brief interview, which included the MoCA (Montreal Cognitive Assessment) test used to control for cognitive status^22^. Only individuals with MoCA score ≥26 (mean group score was 28.4) and without cognitive complaints, psychoactive drug use, head traumas or other conditions that interfere with cognition, were enrolled in the project.

All methods and procedures in this study conform to the Declaration of Helsinki (1964) of the World Medical Association concerning human experimentation. It was approved by the local Ethics Committee (Commission cantonale d′éthique de la recherche sur l′être humain (CER-VD)). To obtain written informed consent, we provided the essential information about the research to each participant.

### EEG recording and pre-processing

The experimental protocol started with interviewing a subject (see Subjects), followed by placing and fitting the EEG cap on the subject’s head. During the recording session (1.5–2.0 hours), subjects were seated in a comfortable chair and instructed to stay awake with eyes closed (Resting state with Eyes Closed, REC). The four REC segments (REC1, REC2, REC3, REC4) alternated with the resting state with eyes open or else with the hand movement task and 2 haptic cognitive tasks. To keep the subjects in a stable awake state over the entire recording session, we alternated the task and REC conditions such that an uninterrupted REC duration could not exceed 3 minutes. Here we report only REC data, which consist of 4 concatenated periods about 8–10 minutes each.

The EEG data were collected by means of mobile electroencephalograph eegosports using a 64-channel waveguard^TM^ original cap (ANT Neuro b.v., Enschede, The Netherlands). The recordings were made with a CPz reference and a high-cutoff filter set to 100 Hz. The electrode impedances were kept under 30 kΩ (10 kΩ in most cases), which was well below the recommended maximum of 50 kΩ for high-impedance eego amplifiers.

The signals were digitized at a rate of 500 samples/s with a 24-bit analog-to-digital converter. They were band-pass filtered from 1 to 45 Hz using a zero-phase filter and edited offline. The artifactual segments were identified by a thorough visual inspection and removed. The signals from mastoid channels were removed from all recordings because of excessive noise in many EEGs. For further analysis, the EEG signals from the remaining 62 EEG channels were re-referenced against the common-average reference.

### General design of EEG analysis

**Figure 1:**
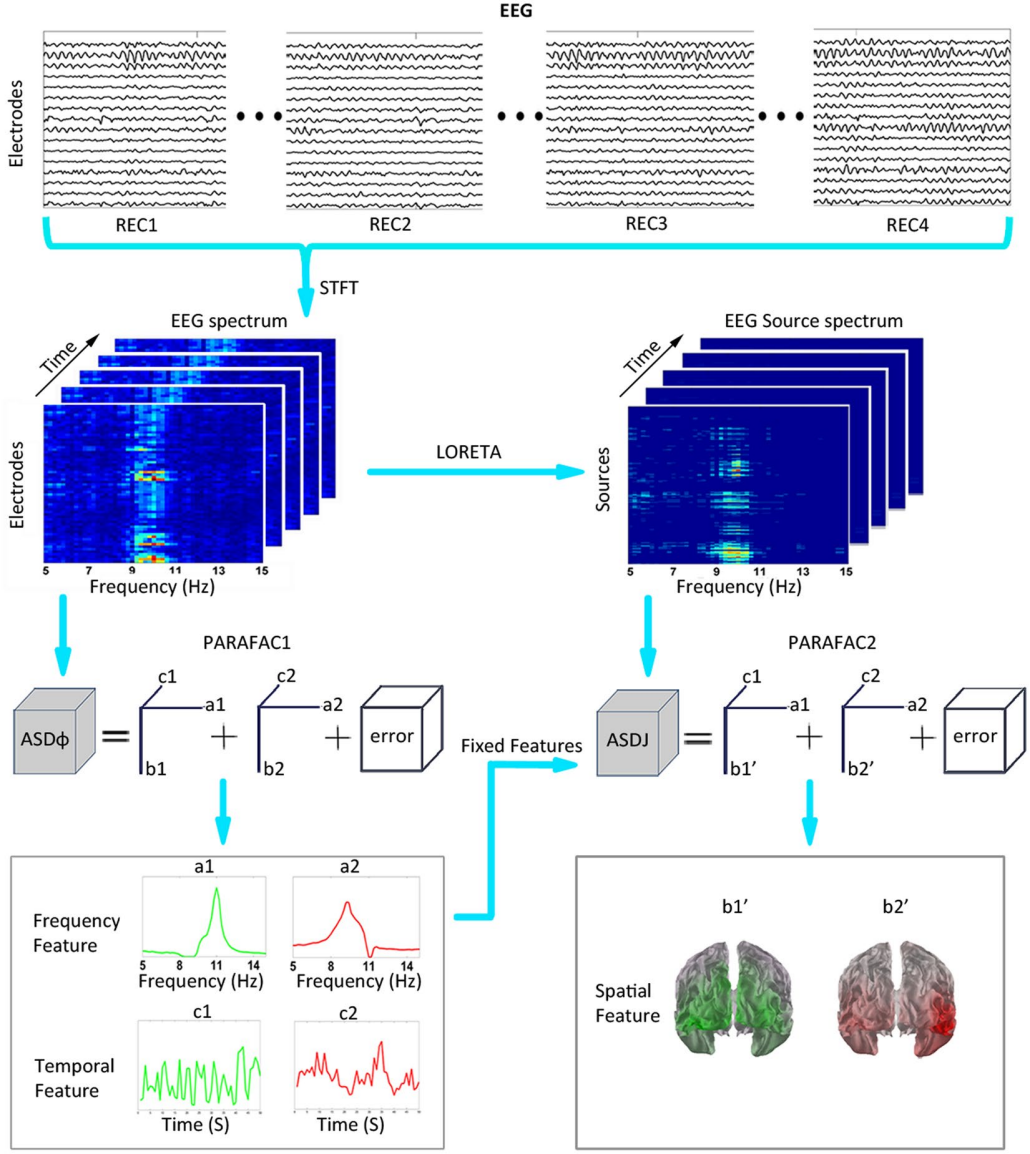
Extraction of alpha rhythm components (ARCs) from EEG signal. The top row shows 4 EEG segments from corresponding REC periods in a single subject. To extract the time-varying EEG power spectrum matrix, Short-Time Fourier Transformation (STFT) was applied to these concatenated EEG time series. Next, the obtained 3-way amplitude spectrum matrix was used as an input for PARAFAC1 to extract the frequency and temporal features of the ARCs. This figure shows a PARAFAC model with two components, where a1 and a2 refer to the frequency features, c1 and c2 indicate temporal features (left bottom panel), and b1 and b2 represent the spatial features of the ARCs in the electrode space (b1 and b2 are not shown, since they were not used for further analysis). To extract the sources of each ARC (b1’ and b2’, right bottom panel), the EEG source spectrum was calculated with the LORETA method. Then, PARAFAC2 was applied to the source amplitude spectrum using fixed frequency (a1, a2) and temporal (c1, c2) features obtained in the sensor-space PARAFAC1. See Methods for details.

A straightforward approach to the analysis of ARCs would be a decomposition of the source-space EEG spectrum by a 3-way parallel factor analysis (PARAFAC), which would produce information about frequency, spatial, and temporal features of ARCs. The main steps would be: calculating the sensor-space EEG spectrum, estimating the source spectrum and, finally, applying PARAFAC to the source spectrum. However, since PARAFAC is more robust for processing data with a higher signal-to-noise ratio and a smaller number of parameters to estimate, we first applied the method to the sensor-space EEG spectrum (PARAFAC1). That allowed us to obtain reliable estimates of the frequency and temporal features of ARCs and to use these estimates as fixed parameters in the application of PARAFAC to the amplitude spectrum of the signals in the source space (PARAFAC2). Figure 1 shows the actual pipeline of our analysis: (1) calculating the EEG spectrum, (2) applying PARAFAC in the sensor space, (3) estimating the source EEG spectrum, and (4) applying PARAFAC in the source space. The following paragraphs provide the necessary details of all the analysis steps.

### Spectral analysis of EEG

We applied a time-frequency analysis to the concatenated EEG recordings from 4 REC periods (Fig. 1) using discrete Short-Time Fourier Transform (STFT) of the time-windowed data. If we consider EEGs as a matrix $D\in {R}^{{N}_{m}\times {N}_{\tau }}$, where R refers to real numbers, *N*_*m*_ is the number of channels, and *N*_*τ*_ is the number of time points over all available REC periods, then the STFT of EEG at a channel *m* is calculated as

1 $${\phi }_{m,f,t}=\frac{1}{L}\sum _{\tau =1}^{{N}_{\tau }}D(m,\tau )W(\tau -t){e}^{-j2\pi f\tau },\,\phi \in {C}^{{N}_{m}\times {N}_{f}\times {N}_{t}}$$

where *W*(*τ* − *t*) is a windowing function of length *L* centered at time *t*, *N*_*f*_ is the number of discrete frequencies, *N*_*t*_ is the number of time windows, and *C* denotes complex numbers. The choice of 5 second Hann windows *W* with 50% overlap resulted in *N*_*t*_ = 781 ± 100 (mean ± standard deviation) per subject, a 0.2 Hz frequency resolution of the spectral data (*df*), and *N*_*f*_ = 51 for the 5–15 Hz extended alpha range that was considered in our analysis.

The cross-spectrum density matrices of scalp EEGs were calculated as

2 $$CSD{\phi }_{f,t}=\frac{2}{(\bar{W{(\tau )}^{2}}).df}.{\phi }_{f,t}.{\phi }_{f,t}^{\ast },\,\,CSD{\phi }_{f,t}\in {C}^{{N}_{m}\times {N}_{m}}$$

where ${\phi }_{f,t}\in {C}^{{N}_{m}\times 1}$ is the STFT of all sensors at time *t* and frequency *f*, and $\frac{2}{(\overline{W{(\tau )}^{2}})}$ is the cross-spectrum normalization factor, with $\overline{W{(\tau )}^{2}}$ being the average of window value squared (for the Hann window it is 0.375).

The EEG amplitude spectrum (amplitude spectral density, ASD) at frequency *f* and time window *t* was calculated as the square root of the diagonal of *CSDφ*_*f*,*t*_. The 3-way EEG amplitude spectrum matrix $ASD\phi \in {R}^{{N}_{m}\times {N}_{f}\times {N}_{t}}$, with frequency, channel, and time dimensions served as the input to the first PARAFAC analysis.

### Source analysis of EEG

To demonstrate the source-space distribution of ARCs, we used the LOw-Resolution Electromagnetic Tomography (LORETA)^23^. The lead field matrix was computed using the 3-shell Locally Spherical Model with Anatomical Constraints (LSMAC) method for 3005 uniformly distributed sources located in the gray matter of the MNI (Montreal Neurological Institute) average brain. The resulting inverse matrix $T\in {R}^{{N}_{s}\times {N}_{m}}$ (where *N*_*s*_ is the number of sources) was down-sampled to 387 uniformly distributed sources in order to reduce the computation time. Using the inverse matrix and sensor-space cross-spectrum density *CSDφ*_*f*,*t*_ the source cross-spectra were computed as

3 $$CSD{j}_{f,t}=T\,.\,CSD{\phi }_{f,t}\,.\,{T}^{T},\,\,CSD{j}_{f,t}\in {C}^{{N}_{s}\times {N}_{s}}.$$

The source amplitude spectrum $ASDj\in {R}^{{N}_{s}\times {N}_{f}\times {N}_{t}}$ was obtained as the square root of the diagonal of *CSDj*.

### Parallel factor analysis

In order to extract the ARCs with their specific frequency, spatial, and temporal features, we employed the PARAFAC method. It is a method for decomposing multi-way data into a small number of hidden variables called components or factors, which explain most of the variance of the original data while reducing its overall dimensionality. Among the multi-way decomposition methods, PARAFAC represents the simplest and the most restricted generalization of bilinear Principle Component Analysis (PCA) to higher order data^24–26^. In contrast to PCA, PARAFAC provides a unique solution, provided the number of components is correctly chosen and the signal-to-noise ratio is appropriate. The method was validated by Bro *et al*.^24, 27^ using simulations and empirical data with known underlying structure. It was applied to EEG for the first time to decompose the topographic components of event related potentials^28, 29^. In more recent applications, PARAFAC was used for the identification of seizure foci in ictal EEG of epileptic patients^30, 31^, the “atomic” decomposition of normal EEG^32^, and for separating the EEG sources on the basis of their phase coupling patterns that vary in space, frequency, and time^33^.

The latter applications of PARAFAC in the EEG area were essentially aimed at introducing this technique as an instrument of EEG decomposition^32^ or proposing an extension of the method^33^ rather than addressing a particular neuroscience problem. The validation of PARAFAC with simulations and its comparisons with other decomposition methods have been reviewed there in detail, and we refer the reader to these reports for an in-depth analysis of these issues^31, 32, 34^. Here we use PARAFAC to show the fine structure of the AR as an individual EEG phenotype. To this end, PARAFAC application is supplemented by a combination of the experimental design features and analysis techniques tuned to the requirements of testing the narrow-band EEG activity, including a high frequency resolution, EEG source reconstruction, long-term and longitudinal EEG recordings.

PARAFAC decomposes multi-way data into a sum of components, which result from a multi-linear production of loading and score vectors. In this study we applied a 3-way (frequency, time, space) PARAFAC decomposition of the EEG spectrum with non-negativity constraints on the loadings to keep the results interpretable. Since in a 3-way PARAFAC analysis loading and score vectors are treated equally, they are not distinguished. To formalize, PARAFAC decomposes the 3-way data matrix *ASDφ* into *K* components as follows:

4 $$ASD{\phi }_{f,m,t}=\sum _{k=1}^{K}{a}_{fk}{b}_{mk}{c}_{tk}+\varepsilon $$

Here, the loading matrices A, B, and C with elements *a*_*fk*_, *b*_*mk*_, *and c*_*tk*_, correspond to the frequency, space (channels or sources), and time modes of the original data. Intuitively, each matrix contributes a “signature” of its corresponding mode to each of the *K* components.

A graphical illustration of this model is presented in Fig. 1. In the PARAFAC analysis, the solution is obtained by the alternating least squares method. This iterative technique estimates the parameters of each mode while assuming that the parameters of two other modes are known. It iterates until convergence. The second and third loading vectors are normalized so that the variance of the data is kept in the first mode. The obtained loading matrices have dimensionless values. To implement PARAFAC, we used the N-way toolbox^35^ in Matlab.

As a result of the first PARAFAC analysis (PARAFAC1 in Fig. 1), for each subject the *ASDφ* in the sensor space was decomposed into a combination of ARCs with specific frequency, channel topography, and time signatures. As an input to the second PARAFAC analysis, we used the 3-way source amplitude spectrum density matrix $ASDj\in {R}^{{N}_{f}\times {N}_{s}\times {N}_{t}}$ with the frequency, source, and time dimensions. The two loading matrices corresponding to the frequency and temporal features had the fixed values calculated in the sensor-space PARAFAC1. Therefore, the source-space model estimated the locations of ARC sources using their predefined frequency and temporal signatures. The source locations were then parcellated according to the Brodmann atlas using the Talairach Daemon software^36^.

### PARAFAC Model verification

To develop a reliable model, we checked the residuals and averages, applied degeneracy tests for the stability and reliability of the model, and performed Core Consistency Diagnostic (CORCONDIA)^27, 29^ using the N-way toolbox. Selection of the appropriate number of components is a critical issue in developing a valid PARAFAC model. Selecting too few components results in a model that is unable to identify the true underlying variables. In contrast, too many components would excessively model noise and/or the true underlying variables would be modeled by correlated components. A number of approaches have been proposed for the selection of a proper number of components including cross-validation, judging residuals, and CORCONDIA^24, 27^. In the preliminary analysis, we used both cross-validation and CORCONDIA, which produced similar results. Ultimately, we decided on CORCONDIA, which has been shown to provide appropriate results in simulations and real datasets with known structure^27^ by testing the model complexity.

The approach comes down to decomposing the data with increasing number of components and calculating the core consistency value, which varies between the maximum of 100% in case of perfect model and 0 or below when the model is invalid. A model with the largest number of components and its core consistency exceeding some threshold is considered as valid. Here, following a proposal by Bro^27^, we set the threshold at 90%.

To remove the noise components, we focused on their frequency and spatial characteristics. The components with flat spectra and non-posterior topography (e.g., those with highest loadings on frontal and anterior temporal regions sensitive to eye- and muscle-related artifacts) were removed from the model. Although this report is focused on the posterior AR, the analysis of whole-head EEG suggested a possibility to identify other ARCs, for instance, the central mu rhythm. For those who are interested in the latter, we provide relevant results in the Supplementary materials.

### Group-level ARC statistics

For each of the 26 participants whose AR was successfully decomposed into at least two ARCs, the highest frequency component was labeled ARC1 and the lowest frequency one ARC2. To compare the source distributions of these two components, we estimated the significant differences of the spatial loadings in the contrasts *ARC1* > *ARC2* and *ARC2* >* ARC1* by means of the one-sided cluster-based permutation test^37^. This non-parametric method extracts the largest significant cluster of sources using permutation test and cluster-based statistics to correct for multiple comparisons.

For each subject, the loading vectors of the source-space PARAFAC2 components were normalized so that their maximum values were set to 1, and, for each source, we computed the one-sided *t*-statistics by comparing the normalized values of the two components. Then, based on spatial adjacency, we selected the clusters of sources with *t*-values above the 99th percentile of the *t*-distribution, and calculated cluster-level statistics as the sums of the *t*-statistics for all sources within each cluster. The ARC labels were permuted 5000 times, and, for each permutation, the largest cluster-level statistic was selected, thus providing a random distribution of the largest cluster-level statistics. Finally, the *P*-value for the largest cluster in the non-permuted data was computed as the number of cluster-level statistics in the random distribution greater than the non-permuted cluster-level statistic divided by the number of permutations.

### Analysis of ARC replicability

To evaluate the temporal stability of the AR structure over a single recording session (1.5–2 hours), for the REC1, REC2, REC3, and REC4 periods of all the subjects with 2 or 3 ARCs, we computed the mean temporal loadings of ARC1 and ARC2 normalized so that the maximum loading was set to 1. To check for possible task effects on the structure of the resting-state AR, we applied a group-level 1-way repeated-measures ANOVA with *task* as a factor (4 levels) and *temporal loadings* of an ARC averaged over each of the REC periods as a dependent variable.

To estimate the source and frequency stability of the AR structure at a time scale of 2 to 13 months, we compared the concatenated data from four REC segments between two separate sessions in 5 subjects available for follow-up. The similarity between loading vectors of ARCs obtained from two different EEG sessions was calculated using Tucker Congruence Coefficients (TCC) as

5 $$TCC=\frac{{\sum }_{k}{x}_{k}.{y}_{k}}{\sqrt{{\sum }_{k}{x}_{k}^{2}.{\sum }_{k}{y}_{k}^{2}}}$$

where *x*_*k*_ and *y*_*k*_ are the elements of loading vectors of a component extracted from the two PARAFAC models. TCC for the non-negative loadings varies between 0 and 1. The TCC value of >0.85 shows highly similar loadings and the TCC value of >0.95 points to nearly identical loadings. We compared loading vectors from spatial and frequency modes.

## Results

### Decomposition of individual AR with PARAFAC

**Figure 2:**
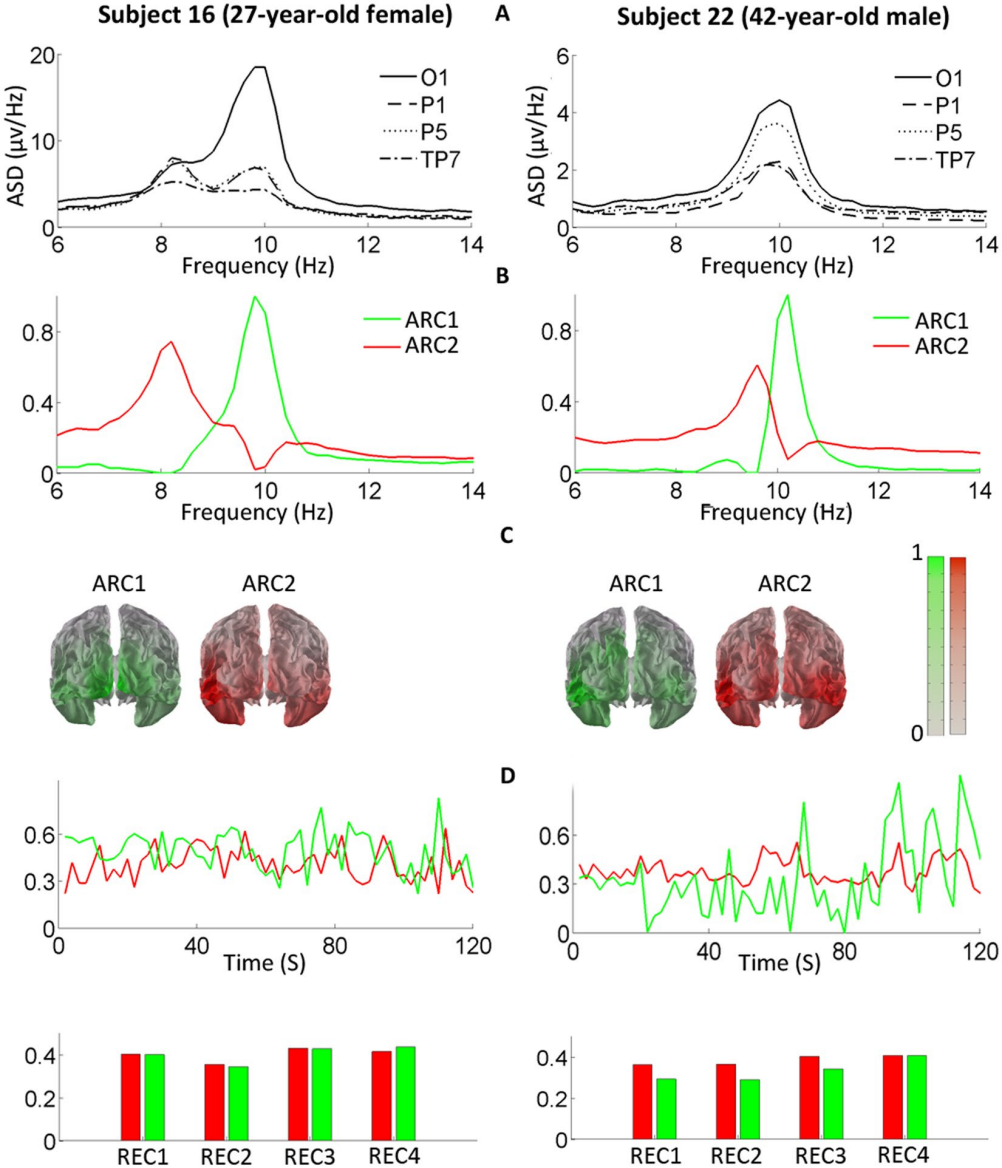
Individual examples of two-component structure of alpha rhythm. (**A**) The amplitude spectrum densities (ASD) of an EEG from posterior electrodes (O1, P1, P5, and TP7 according to the Extended 10/20 System) are presented in the conventional alpha frequency range. (**B**) The frequency loadings of the two ARCs extracted with PARAFAC1. (**C**) The normalized source distributions of the ARCs (spatial loadings of the components) are rendered on the average MNI brain (posterior view). Color indicates loading values: the higher the color intensity, the higher the loadings. (**D**) Temporal loadings of ARC1 (green lines and bars) and ARC2 (red) are shown over representative 3-minute periods (upper row) and as averages over the 4 REC periods (bottom row). The temporal fluctuations of the two components’ loadings are not correlated and both ARCs are well present over the whole EEG session (1.5–2 hours). The dimensionless loading values (y-axes in **B** and **D**) are normalized to the maximum value of each loading matrix.

**Figure 3:**
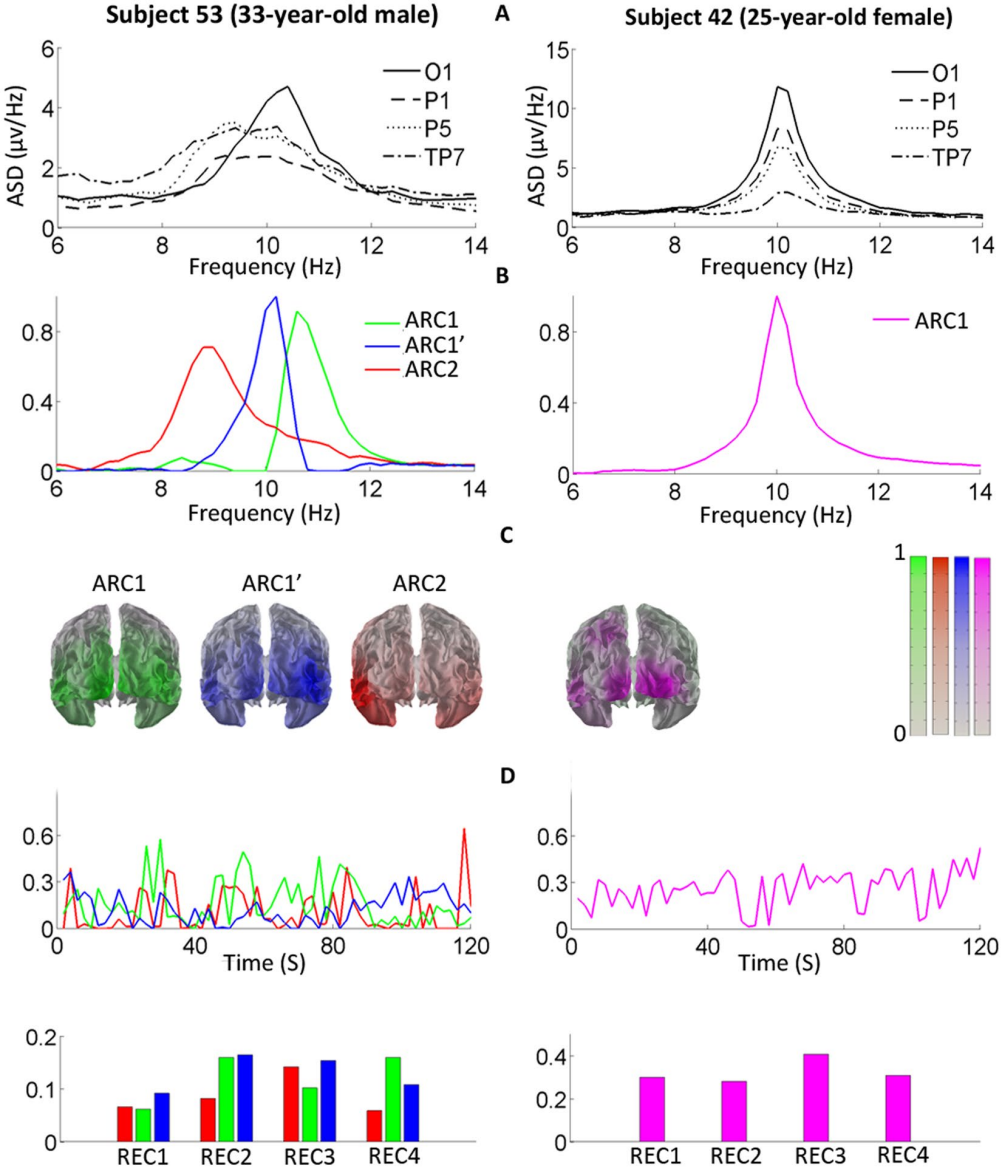
Individual examples of three- and one-component structures of alpha rhythm. All designations are as in Fig. 2. See Results for details.

**Figure 4:**
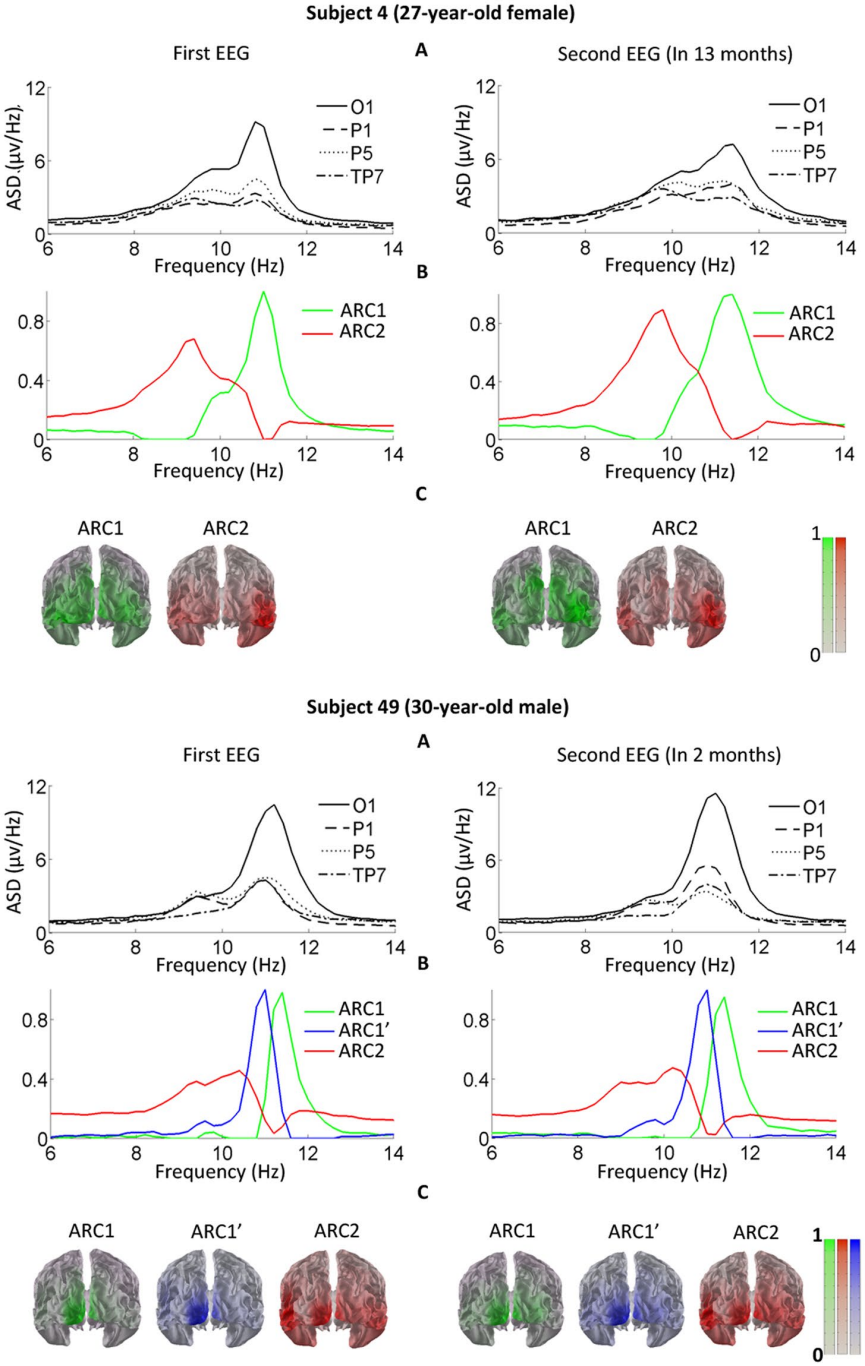
Follow-up illustrates individually stable multi-component structure of alpha rhythm. The representative examples show the component structure of the alpha rhythm in subject 4 with two ARCs and subject 49 with three ARCs, based on two EEG recordings separated by several months. All designations are as in Fig. 2.

The individual amplitude spectra of the common-average-referenced resting-state EEGs suggested that in many subjects more than one rhythm in the alpha range could be recorded from the posterior sensors (Figs 2A, 3A and 4A). Indeed, the decomposition with the PARAFAC method showed that 26 out of 29 subjects had more than one ARC. The typical structure of the AR band, observed in 24 participants (83%) involved 2 ARCs (Fig. 2B). In two thirds of these subjects, the source distributions of the two ARCs were markedly different (Fig. 2C, subject 16; Fig. 4C, subject 4), while in the remaining third the two ARCs had significant overlap of their spectral signatures in the alpha band and similar source distributions (Fig. 2C, subject 22).

The sources of the higher-frequency ARC (ARC1), with its peak frequency around 10 Hz, dominated in the occipito-parietal regions of the neocortex, while the sources of the lower-frequency (around 9 Hz) ARC2 were dominant in the occipito-temporal regions. The maximum loading values of ARC1 were located in the dorsal BA18/19 close to the midline or in the neighboring BA20 in all but 2 subjects, in whom they were localized to BA37. The maxima of ARC2 were located in BA37 (fusiform gyrus) or in the neighboring sites of BA19 or BA20 in all but one subject, in whom the maximum was found in BA18. This 2-component structure was present in both sexes and across the whole age spectrum of our sample (20–45 years).

In 2 male subjects, we found a more complex AR structure represented by 3 ARCs (Fig. 3, Subject 52; Fig. 4, Subject 49). These included the low-frequency occipito-temporal ARC (similar to ARC2 in the 2-component AR) and the two high-frequency ARCs (peak frequency ≥10 Hz) with predominantly occipito-parietal topography, closely resembling ARC1. These 3 ARCs manifested co-existence and individual behavior over the studied temporal intervals (Fig. 3D). The ARs of the remaining 3 subjects could not be decomposed by our analysis (Fig. 3, Subject 42). In these cases, the single ARC had a peak frequency of at least 10 Hz and originated from posterior sources that varied among the subjects, making it difficult to identify the component as any of the previously described ARCs.

### Individual ARC replicability

Throughout a single recording session, the AR structure was relatively stable in all the subjects with 2 or 3 ARCs (Figs 2D, 3D and 4D). Specifically, in this group, the mean temporal loadings for ARC1 in 4 REC periods were 0.35–0.38 and for ARC2, 0.37–0.40. The repeated-measures ANOVA showed no significant modulation of the temporal loadings of ARC1 or ARC2 by the tasks.

To estimate the stability of the AR structure on a large time scale, we compared spatial and frequency loadings from baseline and follow-up EEG recordings of 5 subjects. In all these subjects, ARC1 showed stability of its spatial and frequency features over the two recordings (TCC > 0.85). The spatial loadings of ARC2 also remained stable in all the subjects. In contrast, the frequency loadings of ARC2 were very similar only in 3 subjects, whereas 2 others showed less similarity (0.75 < TCC < 0.80). In subject 49 (Fig. 4) with a 3-component AR structure, the spatial and frequency loadings of the 3rd component were practically equal in the two recordings (TCC > 0.94).

### Group-level analysis of AR components

**Figure 5:**
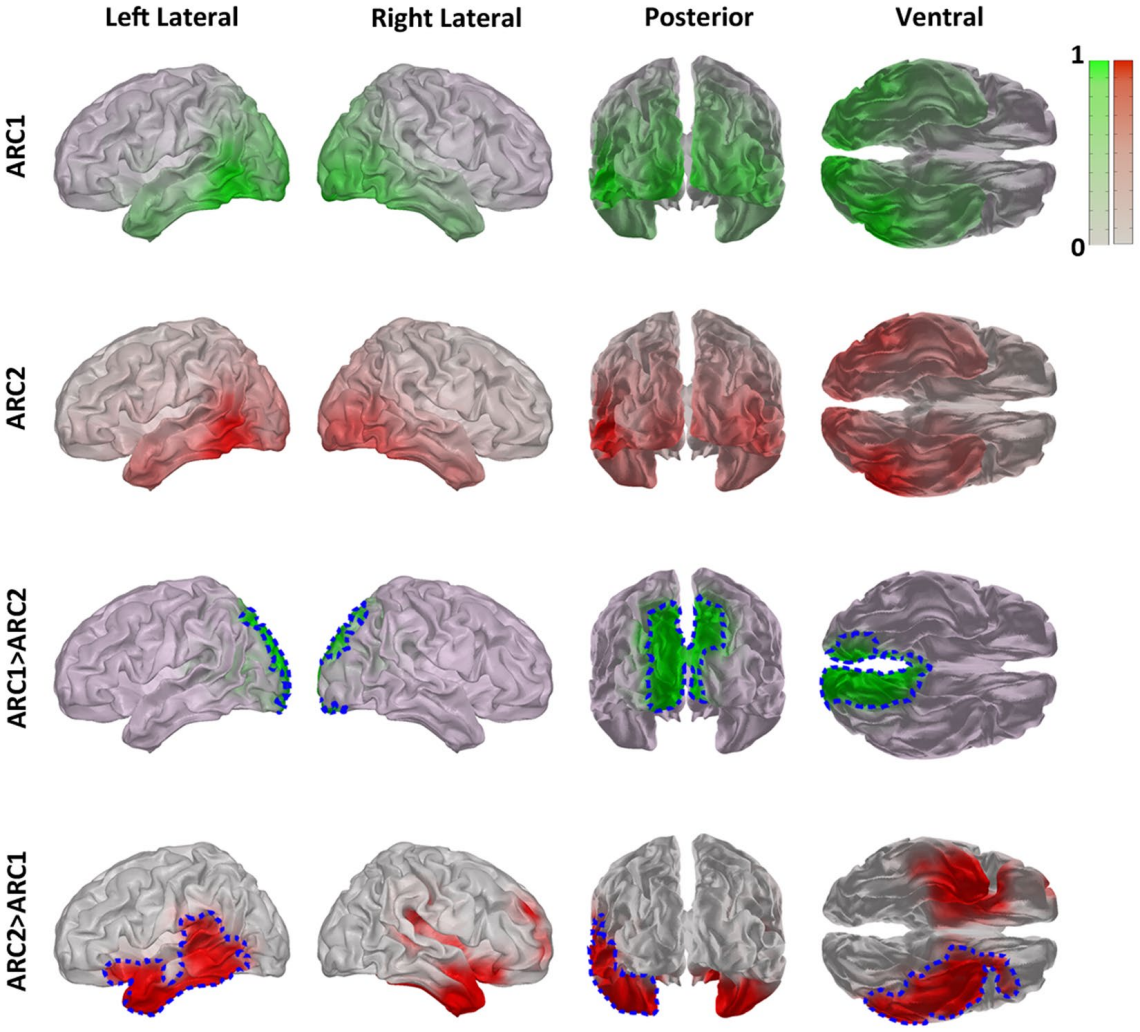
Group level statistics shows different sources of alpha rhythm components. Top row: Normalized group-averaged spatial loadings of ARC1 rendered on the average MNI brain are shown in four views. Second row: Normalized group-averaged spatial loadings of ARC2. Third and fourth (bottom) rows represent the results of uncorrected t-test and cluster-based permutation test. The green color shows the regions where ARC1 has significantly higher loadings than ARC2 (uncorrected), whereas the red cluster shows the locations where ARC2 has significantly higher loadings than ARC1 (uncorrected). The areas bordered by dotted blue line indicate significant clusters that survived correction for multiple comparisons. See Results for details.

Since the posterior AR reveals at least 2 distinct components in 90% of our subjects, one can give a group-level description of the 2-component structure of AR. Specifically, ARC1 has a peak frequency of 10.4 ± 0.6 Hz (mean ± standard deviation) and shows higher loading values (ARC1 > ARC2, *P* = 0.006) in a large (~16 cm^3^) bilateral cluster (Fig. 5) that includes primary and association visual, parietal, and posterior cingulate cortices (BA7, 17, 18, 19, 23). In contrast, ARC2 with a peak frequency of 9.4 ± 0.7 Hz has significantly higher loading values (ARC2 > ARC1, *P* = 0.041) for the left-hemisphere cluster (~7 cm^3^) mainly distributed in the inferior temporal and fusiform gyri (BA20, 36, 37), whereas the right-hemisphere cluster did not survive corrections for multiple comparisons with the cluster-based permutation test.

## Discussion

As outlined in the Introduction, heterogeneity of the posterior AR has been assumed but rarely explicitly tested, and only with two-dimensional decomposition techniques, which left the ARCs unresolved in one of the three EEG dimensions^20, 21^.

Here, with the multidimensional PARAFAC technique, which takes into account the spatial, frequency, and temporal properties of EEG, we demonstrate that a typical structure of the resting-state posterior AR consists of 2 components, originating from the occipito-parietal (ARC1) and occipito-temporal (ARC2) cortices.

Previous EEG/MEG studies consistently localized the equivalent current dipoles (ECDs) of the posterior AR along the brain midline in the parieto-occipital and, to a lesser extent, in the calcarine sulci^38–43^. The EEG/MEG signals were band-pass filtered and an ECD was computed every several milliseconds, resulting in cortical clusters of ECDs. Band-pass filtering of raw EEG/MEG signals within a frequency range spanning the entire alpha band makes problematic the resolution of sources co-existing at neighboring frequencies. This explains why the dominant occipito-parietal component was overemphasized in the literature, while the weaker occipito-temporal component remained under-reported.

The rare reports that addressed the component structure of the posterior AR either did not localize the ARC sources^20, 21, 44^ or localized one of the components to the temporal rather than the occipito-temporal region^45^. Srinivasan and colleagues showed that in MEG both occipital and temporal sensors detect strong ARs, while in the EEG of the same subjects temporal sensors record a much weaker AR than occipital ones. Besides, the temporal AR in MEG had lower peak frequencies than the occipital AR, while this difference was not obvious in EEG. These findings share important features with our results: the occipito-temporal ARC2 reported here is usually weaker and has a lower peak frequency than the occipito-parietal ARC1, and their separation without a decomposition technique is challenging (Figs 2A, 3A and 4A). However, in Srinivasan’s study, the sources of the temporal AR were only broadly defined by the sensor locations covering the entire convexity of the temporal lobe, making it unclear to what extent the EEG-based ARC2 that we describe is homologous to the low-frequency AR recorded by MEG.

The recently reported decomposition of MEG activity by Independent Component Analysis in the cortical source space revealed occipitо-parietal 10 Hz components in all the subjects, and temporal 8–10 Hz components in some of them^45^. The sources of the latter were localized to the superior temporal cortex, suggesting that these components have a different origin from the EEG-based ARC2, which we localized to the fusiform gyrus and neighboring sites. Considering that the temporal AR was mainly recorded with MEG, localized to the midtemporal region, and never reported in the majority of the tested subjects, we conclude that, in previous EEG studies, the occipito-temporal ARC2 was overlooked or not accurately differentiated from the temporal auditory AR.

A prerequisite for AR “visibility” in EEG are layers of radial dipoles occupying cortical territories from several square millimeters to several square centimeters that generate coordinated rhythmic activity^1^. Classical intracranial human studies showed that a strong continual AR occurs over the convexity of the parietal, posterior temporal, and occipital cortices of an exposed brain^46, 47^, leaving the issue of its possible heterogeneity unresolved. This question was examined in a recent electrocorticographic study of epileptic patients, which found two widespread posterior clusters of spontaneous oscillations with dissimilar spatial and frequency features^48^. A relatively narrow cluster peaking at 10 Hz was limited to the occipital and parietal regions, whereas a broad cluster peaking at 7 Hz was mainly located in the occipital and inferior temporal cortices. Considering the EEG slowing in epilepsy^49, 50^, the broad occipito-temporal cluster can be tentatively matched to ARC2.

Spontaneous alpha-range oscillations with their peak frequencies and functional properties distinct in the ocipital and inferotemporal cortices were also recorded in awake macaques^51, 52^. In the occipital areas, pyramids of layer 5 were driving the oscillations, whereas in the inferotemporal cortex, pyramids of layers 2/3 served as a pacemaker (ibid). Since human BA37/19 (lateral occipital complex), where we localized the low-frequency ARC2, is a homolog of the macaque inferotemporal cortex^53^, it is conceivable that, in humans, the laminar mechanisms of the occipito-temporal ARC may also differ from occipito-parietal ones. The fact that the occipito-temporal ARC2 is less powerful than the occipito-parieatal ARC1 favors this suggestion: based on the animal model, a smaller contribution of ARC2 to the surface EEG can be expected due to the tendency of radially distributed dendrites, inherent in the temporal cortices, to form a closed field that falls off faster than a radial dipole configuration typical for the occipital cortex^54, 55^.

In the two subjects with a three-component AR structure, the low-frequency ARC2 was found in the occipito-temporal cortices, whereas the two high-frequency ARCs were localized to the occipito-parietal regions. This finding closely resembles observations by^40, 43, 56^. In^43^, the rhythms peaking around 10 Hz formed two clusters, with the stronger one located in the parietal region and the weaker one in the occipital areas. Here, with the extremely limited number of subjects exhibiting this AR structure, the results of the source reconstruction did not allow us to separate the sources of these high-frequency ARCs. The uncommonness of this configuration in a resting state does not rule out its higher incidence under other conditions. Indeed, as many as three posterior clusters of ARCs with distinct spectral peaks were identified in the subjects involved in a visuo-spatial task^57^.

Repeated observations of the AR show that its frequency and spatial characteristics are subject-specific and manifest the highest heritability among conventional EEG frequency bands^4–6, 58^. These studies considered the AR as a unitary rhythm and, therefore, were biased in favor of the dominant high-frequency occipito-parietal component. To our best knowledge, our study is the first report on the replicability of separate ARCs over weeks or months. Importantly, we estimated not only frequency features of the ARCs, but also their cortical topography. The source distributions of both components as well as the frequency of ARC1 remained highly stable, whereas the ARC2 frequency showed somewhat higher variability, suggesting state-dependent effects on this component.

While the long-term stability of the AR structure remains to be confirmed in adults, the AR “signature”, i.e., its unique component structure, has a good chance to be an individual characteristic, as suggested by an early longitudinal study of healthy children^59^. These authors examined EEG spectra with a high frequency resolution of 0.1 Hz that allowed the differentiation of the posterior AR into 2 components with distinct topographies, peak frequencies, and reactivities. These individual AR structures, found in the EEGs of 7-year-old children, proved to be well-preserved in the follow-up recordings 4 years later.

## Implications for human neuroscience

According to our analysis, in healthy adults the posterior resting-state AR typically consists of two distinct, occipito-parietal and occipito-temporal, components. The cortical topography, individual stability, and similarity to the primate alpha-band organization of this 2-component AR structure link it to the functional modules of the posterior brain, specifically, to the dorsal (ARC1) and ventral (ARC2) visual streams. The next step would be an investigation of functional properties of the two ARCs in paradigms involving tasks that differentially engage the dorsal and ventral visual pathways.

Understanding how many rhythms in the alpha range contribute to the posterior surface EEG is essential for clinical neuroscience as an objective basis for the AR segmentation in the frequency domain. Specifically, the uncommonness of mono- and three-rhythmic structure in the resting-state EEG limits segmentation options in cross-sectional studies. The mechanical (not data-driven) subdivision of the AR into *a priori* defined 3 sub-bands, often encountered in the literature, might provide misleading effects, while treating the AR as a single component (also frequently encountered) results in a mixture of effects originating from the components with different properties. It should be emphasized that unmixing the AR components with the PARAFAC technique *per se* does not require a source analysis, i.e., can be used as a decomposition technique in various clinically motivated analyses of surface EEG.

Finally, the interpretation of AR dynamics under various psychiatric and neurodegenerative conditions requires revision in view of the 2-component model, since apparently similar changes can result from different pathological processes selectively affecting AR components. For instance, the posterior AR often “slows down” in patients with obsessive-compulsive disorder, depression, and Alzheimer’s disease^60–62^. Possible scenarios leading to such changes include a reduction in high-frequency ARC power, an increase in low-frequency ARC power, a decrease in frequency of both components, etc. To learn what really occurs, the study of differential dynamics of the alpha rhythm components is essential.

### Data availability statement

The datasets analyzed during the current study are available from the corresponding author on reasonable request.

## Electronic supplementary material

Supplementary Information
