# Author Correction: Fine Structure of Posterior Alpha Rhythm in Human EEG: Frequency Components, Their Cortical Sources, and Temporal Behavior

**DOI:** 10.1038/s41598-018-24609-3

**Published:** 2018-04-25

**Authors:** Elham Barzegaran, Vladimir Y. Vildavski, Maria G. Knyazeva

**Affiliations:** 1 Laboratoire de recherche en neuroimagerie (LREN), Department of Clinical Neurosciences, Lausanne University Hospital and University of Lausanne, Lausanne, Switzerland; 2 Leenaards Memory Centre and Department of Clinical Neurosciences, Centre Hospitalier Universitaire Vaudois and University of Lausanne, Lausanne, Switzerland; 3 Department of Psychology, Stanford University, Stanford, CA USA

Correction to: *Scientific Reports* 10.1038/s41598-017-08421-z, published online 15 August 2017

This Article contains errors in the Reference list. Reference 19 is incorrectly listed as ‘Nolte, G. *et al*. Identifying true brain interaction from EEG data using the imaginary part of coherency. Clinical Neurophysiology 115, 2292–2307 (2004)’. The correct reference 19 appears below as ref.^1^.

Additionally, reference 45 is incorrectly cited in its second instance. The correct reference 45 appears below as ref.^2^.

Consequently, in the Discussion section,

‘The recently reported decomposition of MEG activity by Independent Component Analysis in the cortical source space revealed occipitо-parietal 10 Hz components in all the subjects, and temporal 8–10 Hz components in some of them^45^’.

should read:

‘The recently reported decomposition of MEG activity by Independent Component Analysis in the cortical source space revealed occipitо-parietal 10 Hz components in all the subjects, and temporal 8–10 Hz components in some of them^2^’.
