# Supplementary material for: Fine Structure of Posterior Alpha Rhythm in Human EEG: Frequency Components, Their Cortical Sources, and Temporal Behavior
Source: Sci Rep. 2017 Aug 15;7:8249. doi: 10.1038/s41598-017-08421-z (PMC5557761; doi:10.1038/s41598-017-08421-z)
Supplement: Supplementary file 1 — Supplementary Information [file 41598_2017_8421_MOESM1_ESM.pdf]

Fine Structure of Posterior Alpha Rhythm in Human EEG: Frequency Components, Their Cortical Sources, and Temporal Behavior

Running title: ***Components of Posterior Alpha Rhythm***

Elham BARZEGARAN[1,2], Vladimir Y. VILDAVSKI[3], and Maria G. KNYAZEVA[1,2]

[1] *Laboratoire de recherche en neuroimagerie (LREN), Dept. of Clinical Neurosciences, Lausanne University Hospital and University of Lausanne, SWITZERLAND*

[2]*Leenaards Memory Centre and Dept. of Clinical Neurosciences, Centre Hospitalier Universitaire Vaudois and University of Lausanne, SWITZERLAND*

[3]*Department of Psychology, Stanford University, Stanford, CA, USA*

Correspondence and reprint requests to:
Maria G. Knyazeva
Neuroimaging Research Lab (LREN)
Department of Clinical Neurosciences
Lausanne University Hospital (CHUV)
Bureau MP16 05/559
Rue du Mont-Paisible 16,
Cité Hospitalière CHUV
CH-1011 Lausanne

Email: Maria.Knyazeva@chuv.ch
Phone: +4121 3143231

Supplementary Material

Detecting Mu Rhythm with PARAFAC Decomposition

In surface EEG the mu rhythm is represented by oscillations typically in the upper AR range best recorded from the central and neighboring electrodes at rest [1-4]. Since the mu rhythm originates from the sensorimotor cortex and is attenuated by voluntary movements and somatosensory stimulation, it is classically considered as a sensorimotor analogue of the visual AR [5,6].

In our sample of 29 subjects, we could visually distinguish the mu rhythm in 8 subjects (28%) based on its frequency, location, and reactivity (Fig. S1). However, PARAFAC analysis applied to the whole-head EEG from 62 electrodes failed to detect an ARC with the central or pericentral location in any subject. This result is consistent with previous publications on AR decomposition [7,8]. Both studies did not detect mu rhythm among their AR components. Moreover, in a recent electrocorticographic study, Groppe and colleagues (2013) found two widespread posterior clusters of spontaneous oscillations in the AR range similar to our ARC1 and ARC2, but failed to distinguish the mu thythm in the group analysis, although individual mu peaks were visible over the pre- and postcentral gyri [9].

To understand the reasons for this failure, we selected for further analysis the EEGs from 4 subjects with the best-pronounced mu rhythm. In all of them the mu peak had its spatial maximum at the C3 and C4 locations, a higher frequency, and much lower amplitude than those of the posterior AR. Therefore, the likely reasons of the mu rhythm “invisibility” for decomposition techniques could be its local character, and/or its relatively small amplitude, and/or partial overlap with the posterior AR. To counterbalance for the limited spatial extent of the mu activity, the data from only 15 central and pericentral electrodes (FC1, FC2, FC3, FC4, FCz, C1, C2, C3, C4, Cz, CP1, CP2, CP3, CP4, CPz) were submitted to

PARAFAC. The method failed to extract the mu rhythm as a separate component even with this subset of channels corresponding to the scalp area of mu activity.

In an attempt to compensate for the low amplitude of the mu rhythm, we whitened EEG spectra by dividing the ASD at each frequency by its mean over all the electrodes, thus reducing the amplitude of the widespread posterior AR relative to the amplitude of the more local mu rhythm. The application of PARAFAC to all 62 whitened EEG channels provided wide-band noise components, but still did not reveal an ARC with the mu-rhythm properties. Finally, with the subset of whitened EEG spectra from the 15 central and pericentral channels, the PARAFAC detected the mu rhythm as a separate component in these 4 preselected subjects (Fig. S2).

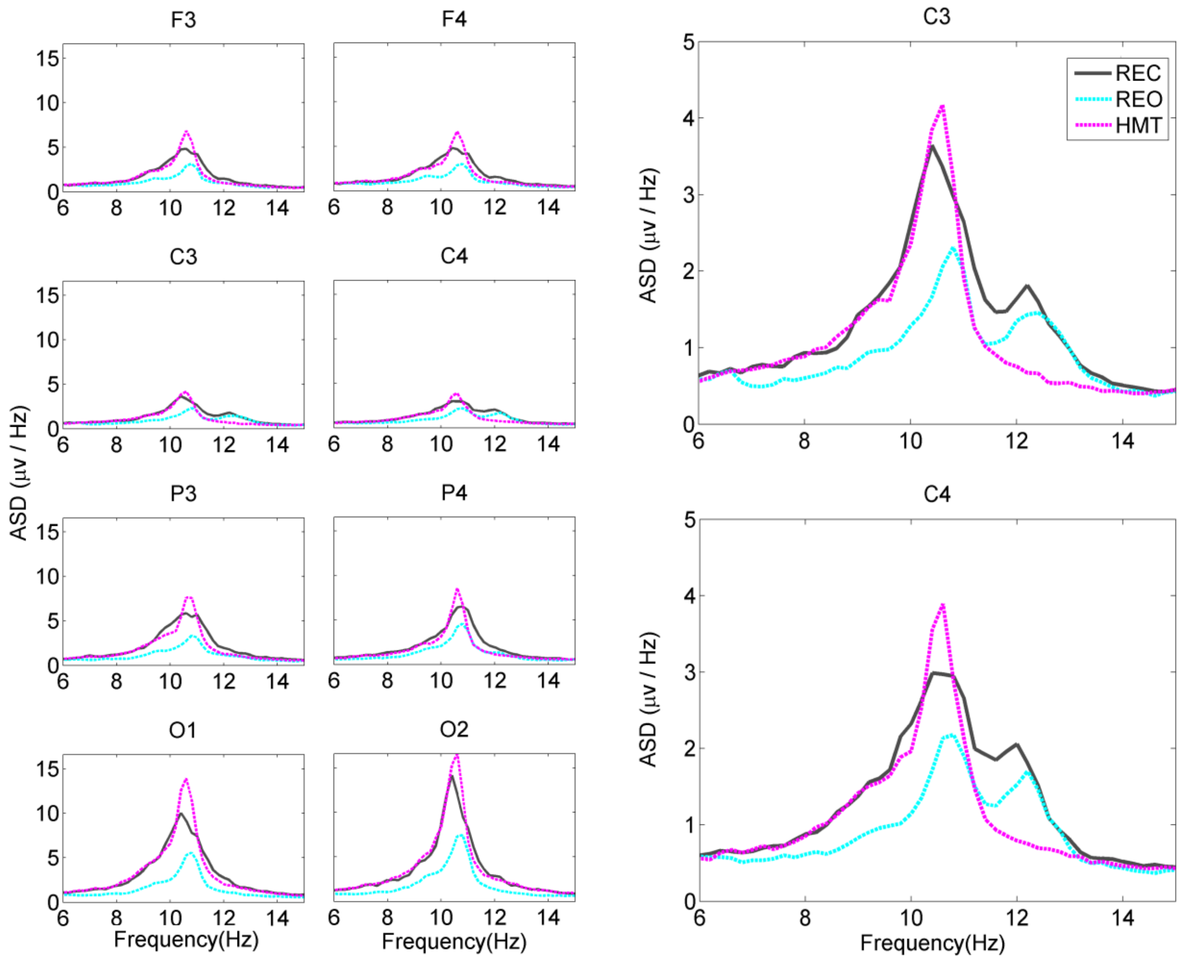

Figure S1. Amplitude spectrum density (ASD) for common-average reference EEG in subject 21 On the left, the ASD from 8 electrodes (10/20 International system) are shown for the resting state with eyes closed (REC), resting state with eyes open (REO), and during a hand movement task (HMT)

performed with closed eyes. On the right, the magnified ASD from the two central electrodes is presented. The mu rhythm with a peak at about 12 Hz is seen in the REC and REO conditions, while being suppressed during the HMT.

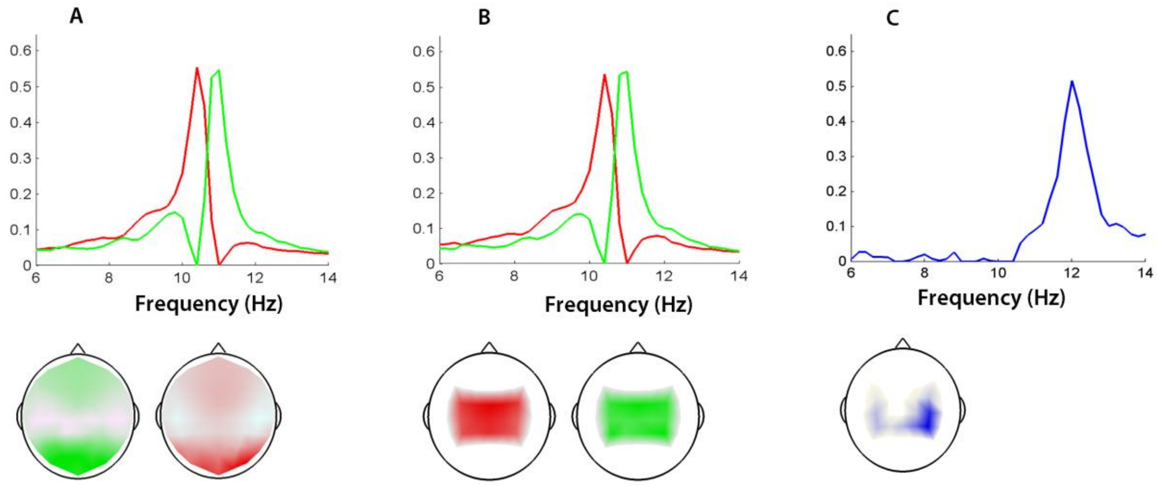

Figure S2. PARAFAC analysis for subject 21 on 62 electrodes (A), 15 electrodes (B), and 15 electrodes with whitened spectra (C)

In the top row, the frequency loadings of ARC1 (green), ARC2 (red), and the mu rhythm (blue) are presented. In the bottom row, the spatial loadings of PARAFAC models are shown in the sensor-space EEG. Other designations are as in Figure 2.

Therefore, in the analyses, tuned to the decomposition of the posterior AR, the mu rhythm remains untraceable due to its local appearance, relatively small amplitude, and, possibly, its frequency overlapping with the posterior AR, which obscures the weaker mu rhythm. Apparently, its detection requires special adjustments of decomposition techniques and targeted optimizations of experimental designs, e.g., using open-eyes conditions, which reduce the high-amplitude posterior AR overshadowing the mu activity [10] (see Fig. S1).

References

1 Andrew, C. & Pfurtscheller, G. On the existence of different alpha band rhythms in the hand area of man. *Neuroscience letters* **222**, 103-106 (1997).

2 Cuevas, K., Cannon, E. N., Yoo, K. & Fox, N. A. The infant EEG mu rhythm: methodological considerations and best practices. *Developmental Review* **34**, 26-43 (2014).

3 Hari, R. & Salmelin, R. Human cortical oscillations: a neuromagnetic view through the skull. *Trends in neurosciences* **20**, 44-49 (1997).

4 Kuhlman, W. N. Functional topography of the human mu rhythm. *Electroencephalography and clinical neurophysiology* **44**, 83-93 (1978).

5 Salenius, S., Schnitzler, A., Salmelin, R., Jousmäki, V. & Hari, R. Modulation of human cortical rolandic rhythms during natural sensorimotor tasks. *NeuroImage* **5**, 221-228 (1997).

6 Salmelin, R. & Hari, R. Characterization of spontaneous MEG rhythms in healthy adults. *Electroencephalography and clinical neurophysiology* **91**, 237-248 (1994).

7 Chiang, A., Rennie, C., Robinson, P., Van Albada, S. & Kerr, C. Age trends and sex differences of alpha rhythms including split alpha peaks. *Clinical Neurophysiology* **122**, 1505-1517 (2011).

8 Lodder, S. S. & van Putten, M. J. Automated EEG analysis: Characterizing the posterior dominant rhythm. *Journal of neuroscience methods* **200**, 86-93 (2011).

9 Groppe, D. M. *et al.* Dominant frequencies of resting human brain activity as measured by the electrocorticogram. *NeuroImage* **79**, 223-233 (2013).

10 Makeig, S. *et al.* Dynamic brain sources of visual evoked responses. *Science* **295**, 690-694 (2002).
